# Moderate Prenatal Alcohol Exposure Enhances GluN2B Containing NMDA Receptor Binding and Ifenprodil Sensitivity in Rat Agranular Insular Cortex

**DOI:** 10.1371/journal.pone.0118721

**Published:** 2015-03-06

**Authors:** Clark W. Bird, Felicha T. Candelaria-Cook, Christy M. Magcalas, Suzy Davies, C. Fernando Valenzuela, Daniel D. Savage, Derek A. Hamilton

**Affiliations:** 1 Department of Psychology, University of New Mexico, Albuquerque, New Mexico, United States of America; 2 Department of Neurosciences, University of New Mexico, Albuquerque, New Mexico, United States of America; Nathan Kline Institute for Psychiatric Research and New York School of Medicine, UNITED STATES

## Abstract

Prenatal exposure to alcohol affects the expression and function of glutamatergic neurotransmitter receptors in diverse brain regions. The present study was undertaken to fill a current gap in knowledge regarding the regional specificity of ethanol-related alterations in glutamatergic receptors in the frontal cortex. We quantified subregional expression and function of glutamatergic neurotransmitter receptors (AMPARs, NMDARs, GluN2B-containing NMDARs, mGluR1s, and mGluR5s) by radioligand binding in the agranular insular cortex (AID), lateral orbital area (LO), prelimbic cortex (PrL) and primary motor cortex (M1) of adult rats exposed to moderate levels of ethanol during prenatal development. Increased expression of GluN2B-containing NMDARs was observed in AID of ethanol-exposed rats compared to modest reductions in other regions. We subsequently performed slice electrophysiology measurements in a whole-cell patch-clamp preparation to quantify the sensitivity of evoked NMDAR-mediated excitatory postsynaptic currents (EPSCs) in layer II/III pyramidal neurons of AID to the GluN2B negative allosteric modulator ifenprodil. Consistent with increased GluN2B expression, ifenprodil caused a greater reduction in NMDAR-mediated EPSCs from prenatal alcohol-exposed rats than saccharin-exposed control animals. No alterations in AMPAR-mediated EPSCs or the ratio of AMPARs/NMDARs were observed. Together, these data indicate that moderate prenatal alcohol exposure has a significant and lasting impact on GluN2B-containing receptors in AID, which could help to explain ethanol-related alterations in learning and behaviors that depend on this region.

## Introduction

Exposure to alcohol during nervous system development results in deficits in behavior and cognition [1–10]. Recent studies estimate that between 2% and 5% of children in the United States could be diagnosed with fetal alcohol spectrum disorders (FASDs) [11], which include Fetal Alcohol Syndrome (FAS), partial FAS (pFAS), and alcohol-related neurodevelopmental disorders (ARNDs) [12]. While exposure to high levels of alcohol can cause the facial dysmorphias characteristic of FAS, more moderate prenatal ethanol exposure can cause persistent cognitive deficits and other adverse neurobehavioral consequences in the absence of birth defects in humans with FASDs [13–15] as well as in model organisms exposed to ethanol during development [16]. A relatively high percentage (5% to >30%) of women in the United States report consuming some alcohol while pregnant [17–22], and nearly 50% of women report drinking prior to recognition of pregnancy [23–25], which may increase the general incidence of FASDs despite efforts to increase awareness of the risks of drinking to the developing fetus. Maternal drinking rates in countries outside North America are even higher, with 38–40% of Brazilian women [26, 27], 43% of South African women [28], 45% of Spanish women [29], 59% of Western Australian women [30], 60% of Russian women [31], and 63% of Uruguayan women [32] reporting consumption of alcohol at some point during pregnancy, demonstrating that prenatal alcohol exposure is endemic around the world. Understanding the behavioral and underlying neurobiological effects of moderate alcohol exposure is important given that most FASD cases encompass the less severe forms [33].

Prior work from our laboratory on the neurobiological consequences of moderate prenatal alcohol exposure (PAE) revealed reductions in experience-dependent immediate early gene (IEG) expression in cortical layers II/III in ventrolateral frontal cortex, specifically the agranular insular cortex (AID) and lateral orbital (LO) regions, as well as pre-limbic cortex (PrL) [34, 35] following social interaction. Expression of *c-fos* was reduced in the AID, LO, and PrL, whereas reductions in *activity regulated cytoskeleton-associated protein* (*Arc*) mRNA were limited to AID. Further, decreases in spine density were limited to the apical dendritic fields of layer II/III pyramidal neurons in AID. Dendritic spine density and levels of IEG expression are modulated by excitatory glutamatergic neurotransmission mediated through α-amino-3-hydroxy-5-methyl-4-isoxazolepropionic acid (AMPA), N-methyl-D-aspartate (NMDA), and metabotropic glutamate receptors [36–40]. PAE affects the expression and physiology of these glutamate receptors, as well as GABA [41, 42], dopamine [43, 44], serotonin [45], and opioid [46] receptors in a number of brain regions. Regionally specific glutamate receptor expression in the superficial layers of ventrolateral (AID and LO) and medial (PrL) frontal cortex has not been examined following moderate PAE. Alterations in glutamatergic receptor expression and function may help inform the neurobiological bases of frontal-cortex dependent behavioral deficits in animals following moderate PAE, which have the potential to provide insight into behavioral alterations observed in humans diagnosed with FASDs.

To address this gap in knowledge, the present study was designed to examine expression of ionotropic and metabotropic glutamate receptors following moderate PAE through autoradiographic quantification of tritiated radioligand binding in several fronto-cortical regions including AID, LO, PrL and M1 (primary motor cortex). Interestingly, we did not find differences in AMPA, total NMDA, or metabotropic glutamate receptor (mGluR2/3 and mGluR5) expression anywhere in the frontal cortex in animals exposed to alcohol *in utero*. However, radioligand binding to GluN2B-containing NMDA receptors (NMDARs) was elevated in layer II/III of AID relative to other regions (LO, PrL, and M1) in PAE animals. Based on the radioligand binding information we sought to characterize AMPA and NMDA receptor function, AMPA/NMDA ratios, and multiplicity ratios (a measure of neuronal connectivity) in AID using whole-cell patch-clamp electrophysiology, as PAE-induced alterations in excitatory neurotransmitter receptor activity may be independent of overall receptor expression. We also examined the effects of ifenprodil, a GluN2B specific NMDA receptor negative allosteric modulator, on evoked currents in layer II/III pyramidal neurons in AID based on the elevated GluN2B expression detected in the autoradiography studies.

## Methods

All chemicals were purchased from Sigma-Aldrich (St. Louis, MO) unless otherwise stated. All procedures included in the manuscript adhered to the Public Health Service policy on humane care and use of laboratory animals and were approved by the Institutional Animal Care and Use Committee of the University of New Mexico (IACUC protocol number MCC-101106).

### Rats

Adult (> 90 days old) male and female Long-Evans rats bred at the University of New Mexico Health Sciences Center animal research facility were used. A total of 28 rats (7 males and 7 females from each prenatal treatment condition) were used for quantitative autoradiography measurements and 40 rats (10 males and 10 females from each prenatal treatment condition) were used for acute slice electrophysiology experiments. All rats were pair-housed with a partner of the same sex and prenatal treatment condition on a 12-hr light/dark cycle with food and water available ad libitum. No more than one offspring from each litter and dam were used in these experiments.

### Voluntary Drinking Paradigm

The voluntary drinking paradigm used in this study was previously described by Savage *et al*. [47]. Three- to four-month-old Long-Evans rat breeders (Harlan Industries, Indianapolis, IN) were single-housed in plastic cages at 22°C and kept on a “reverse” 12-hour dark/12-hour light schedule (lights on from 2100 to 0900 hours) with Harlan Teklad rat chow and tap water *ad libitum*. After acclimating to the animal facility for 1 week, all female rats were provided 0.066% (w/v) saccharin in tap water for 4 hours each day from 1000 to 1400 hours. On Days 1 & 2, the saccharin water contained 0% ethanol and on Days 3 & 4 it contained 2.5% ethanol (v/v). On Day 5 and thereafter, the saccharin water contained 5% ethanol (v/v). Daily ethanol consumption was monitored for at least 2 weeks and mean daily ethanol consumption was determined for each female breeder. Breeders that drank 1 standard deviation above or below the group mean were excluded from the study. Remaining females were randomly assigned to either a saccharin control or 5% ethanol drinking group and matched such that the mean pre-pregnancy ethanol consumption by each group was similar. Tap water was available during all phases of the drinking paradigm.

Subsequently, females were housed with proven male breeders until pregnant as evidenced by the presence of a vaginal plug. Female rats did not consume ethanol during the breeding procedure. Beginning on gestational Day 1 (GD1), rat dams were provided saccharin water containing either 0% or 5% ethanol for 4 hours a day. The volume of 0% ethanol saccharin water provided to the controls was matched to the mean volume of saccharin water consumed by the 5% ethanol drinking group. Daily 4-hour ethanol consumption was recorded for each dam. To minimize handling-induced stress, rat dams were weighed once a week during pregnancy and daily weight gains extrapolated to determine daily ethanol consumption. At birth, access to ethanol was discontinued and all litters were weighed and culled to 10 pups. Offspring were weaned at 24 days of age and maintained and pair housed with a same-sex cage mate. In all experiments only 1 animal per litter was used to avoid potential litter effects.

### Maternal Serum Ethanol Levels

A separate set of twelve rat dams was used to determine serum ethanol concentrations. These dams completed the same voluntary drinking paradigm as described above, except that at the end of the 4-hour ethanol consumption period on each of 3 alternate days during the third week of gestation, each rat dam was briefly anesthetized with isoflurane. 100 μl of whole blood was collected from the tail vein and immediately mixed with 0.2 ml of 6.6% perchloric acid, frozen and stored at -2°C until assayed. Serum ethanol standards were created by mixing whole blood from untreated rats with known amounts of ethanol ranging from 0 to 240 mg ethanol/dl and then mixing 100 μl aliquots of each standard with perchloric acid and storing the standards frozen with the samples. Serum ethanol samples were assayed using a modified method of Lundquist and colleagues [48].

### *In Vitro* Autoradiography Assays

Adult rats (≥ 3 months old) were sacrificed by rapid decapitation, brains were dissected, frozen in a dry-ice methanol bath, and stored at -80°C until histological sectioning. Coronal sections (12μm) were collected on a microtome cryostat starting at a point where the anterior aspect of the corpus callosum (forceps minor) is first visible, around Bregma 4.20 mm, corresponding to Plates #8–9 in the stereotaxic atlas of Paxinos and Watson [49]. Nissl-stained sections were used to confirm the appropriate level of sectioning. The sections were thaw-mounted onto pre-cleaned Superfrost-plus microscope slides (Fisher Scientific, Hampton, NH) and stored at -80°C until assay. At the beginning of each assay sections were pre-incubated twice for 15 min each at 4°C in incubation buffer to remove endogenous glutamate and rinsed twice for 5 minutes in incubation buffer.

For AMPA receptor (AMPAR) binding, sections were incubated in buffer (50 mM Tris-HCl, 100 mM KCl, pH 7.4) for 1 hour at 4°C with 20 nM [^3^H]-fluorowillardiine (FW) (American Radiolabeled Chemicals, Saint Louis, MO), in the absence or presence 10 μM quisqualate (non-specific binding). Sections were rinsed twice for 5 sec each at 4°C. For total NMDA receptor binding, sections were incubated in buffer (30 mM Tris-HEPES, pH 7.4) for two hours at 25°C with 5 nM [^3^H]-MK-801 (NEN Life Sciences/Perkin Elmer, Boston, MA), 100 μM l-glutamate and 100 μM l-glycine, in the presence or absence of 100 μM unlabeled MK-801 (non-specific binding). Sections were rinsed twice for 20 min each at 4°C. For quantification of GluN2B-containing receptors (GluN2BRs), sections were incubated in buffer (50 mM Tris-HCL, 100 mM KSCN, pH 7.4) for one hour at 4°C with 6.25 nM [^3^H]-ifenprodil (NEN Life Sciences/Perkin Elmer), 3 μM R(+)-3-(3-hydroxyphenyl)-N-propylpiperidine HCl and 10 μM GBR 12909 (to prevent ifenprodil binding to sigma receptors [50]), in the absence or presence of 3 mM spermine (non-specific binding). Sections were rinsed twice for 5 min each at 4°C. For mGluR_5_ receptor binding, sections were incubated in buffer (30 mM HEPES, 110 mM NaCl, 1.2 mM MgCl_2_, 5 mM KCl, 2.5 mM CaCl_2_, pH 7.4) for 60 minutes at 25°C with 20 nM [^3^H]-MPEPy (gift from Merck & Co., Inc., Whitehouse Station, NJ), in the absence or presence of 100 μM SIB 1893 (non-specific binding). Sections were rinsed twice for one min each at 4°C. For group II/II mGluR binding, sections were incubated in buffer (10 mM KH_2_PO_4_, 100 mM KBr, pH 7.6) for 90 minutes at 4°C with 5 nM [^3^H]-LY 341,495 (American Radiolabeled Chemicals), in the absence or presence of unlabeled 100 μM LY 341,495 (non-specific binding). Sections were rinsed twice for one min each at 4°C. After sections were rinsed, they were quickly dipped in ice-cold distilled water, air-dried under a stream of cool air, and vacuum desiccated overnight. Sections were loaded into X-ray cassettes along with standards containing known amounts of tritium embedded in plastic. Kodak Biomax MR film was apposed to the sections and tritium standards then exposed, developed, fixed, rinsed and dried. Film exposure times were 9 weeks for FW and 8 weeks for MK-801, ifenprodil, LY 341,495 and MPEPy binding.

**Fig 1:**
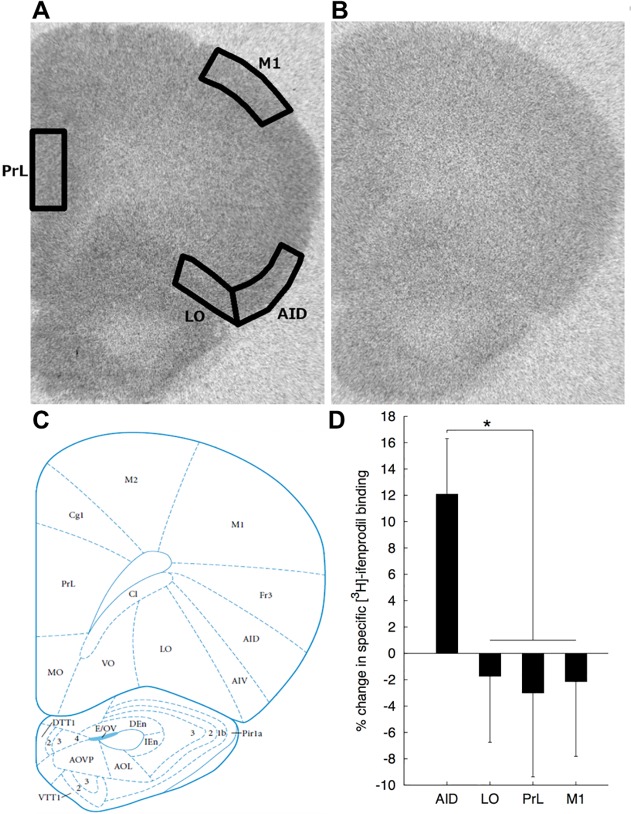
Sampled brain areas for autoradiography densitometry analysis and % change in [^3^H]-ifenprodil binding. **A)** [^3^H]-Ifenprodil binding in a coronal section of rat brain corresponding to Plate #8 (Bregma + 4.2 mm) in the stereotaxic atlas of Paxinos and Watson [49]. Densitometry of [^3^H]-ifenprodil binding was measured in cortical layers I-III in prelimbic cortex (PrL), primary motor cortex (M1), lateral orbital cortex (LO), and agranular insular cortex (AID). The image is overlaid with boundaries of sampled regions. **B)** Image of non-specific [^3^H]-ifenprodil binding. **C)** Plate #8 from the stereotaxic atlas of Paxinos and Watson [49] (Reprinted with permission from Elsevier). **D)** Mean (+SEM) percent change in [^3^H]-ifenprodil binding in AID, LO, PrL, and M1 are shown. Asterisk (*) indicates a significant (p < 0.05) prenatal treatment X region interaction for [^3^H]-ifenprodil binding.

Densitometric analysis was performed using Media Cybernetics Image Pro Plus on an Olympus BH-2 microscope at a total magnification of 3.25X. For each measurement of a tritium standard or sample autoradiogram, a background image was subtracted from the primary image. The background image consists of a sheet of plastic with the same optical density as unexposed film mounted onto a clean glass microscope slide that compensates for uneven field illumination. After background correction, the area of interest was drawn and the average grey level value within the region was determined. The four regions of interest were the AID, LO, PrL and M1 and sampling was limited to the superficial layers (I-III) (**Fig. 1**). The amount of receptor binding in sections, expressed as femtomoles/105 mm^2^, was determined by obtaining grey level measurements over the regions of interest, performing regression analysis of these values on the tritium standard curve and then dividing the resulting value by the specific activity of the radioligand. Grey level measurements of binding on the right and left side of four sections incubated in the absence of excess unlabeled ligand were used to obtain a measure of total receptor binding in each brain region of interest. The right and left side of two sections incubated with excess unlabeled ligand were used to obtain a measure of nonspecific receptor binding. Specific receptor binding is defined as the difference between total and nonspecific receptor binding.

### Electrophysiology

Adult rats (≥ 3 months old) were deeply anaesthetized with ketamine (250 mg/kg body weight) and decapitated. Brains were rapidly removed and placed in ice-cold N-methyl-D-glucamine (NMDG)-based cutting solution composed of (in mM): 92 NMDG, 2.5 KCl, 1.2 NaH_2_PO_4_, 30 NaHCO_3_, 20 HEPES, 25 glucose, 2 thiourea, 3 sodium pyruvate, 5 ascorbic acid, 10 MgSO_4_, and 0.5 CaCl_2_, equilibrated with 95%O_2_/5%CO_2_. Coronal sections (300 μm) were cut with a Vibratome (Technical Products, St Louis, MO, USA). After cutting, slices were transferred to 32°C NMDG cutting solution equilibrated with 95%O_2_/5%CO_2_ for 15 minutes, then transferred to a room-temperature holding solution composed of (in mM): 92 NaCl, 2.5 KCl, 1.2 NaH_2_PO_4_, 30 NaHCO_3_, 20 HEPES, 25 glucose, 2 thiourea, 3 sodium pyruvate, 5 ascorbic acid, 1 MgSO_4_, and 2 CaCl_2_, equilibrated with 95%O_2_/5%CO_2_ for 50 minutes before recording. During recording, slices were superfused with aCSF composed of (in mM): 125 NaCl, 2 KCl, 1.131 NaH_2_PO_4_, 26 NaHCO_3_, 2 CaCl_2_, 1 MgSO_4_, and 0.4 ascorbic acid, continuously bubbled with 95%O_2_/5%CO_2_ at a rate of 2 ml/min and maintained at 32°C. Neurons were visualized with infrared differential interference contrast microscopy. Recordings were made using an A-M systems model 2400 amplifier (Sequim, WA) and digitized at a rate of 10 kHz and filtered at 2 kHz (4 pole Bessel low-pass filter). Patch pipettes (tip resistance 3–5 MΩ) were filled with an internal solution composed of (in mM): 140 potassium D-gluconic acid, 10 HEPES, 1 EGTA, 6 KCl, 0.1 CaCl_2_, 5 MgCl_2_, 4 NaATP, 0.4 NaGTP, and 0.5% biocytin, osmolarity 280–290 mOsm. The holding potential was -70 mV, which was corrected for liquid junction potential (15.2 mV). Recordings were rejected if the access resistance changed by ≥20% during the course of the experiment.

20 rats (5 males and 5 females from each prenatal diet condition) were used to measure spontaneous post-synaptic currents. Spontaneous excitatory post-synaptic currents (sEPSCs) were recorded in the presence of picrotoxin (PTX) (50 μM) to eliminate GABA_A_ receptor-mediated inhibitory neurotransmission. EPSCs were analyzed using the Mini Analysis Program (Synaptosoft, Fort Lee, NJ) to measure amplitude and frequency of spontaneous synaptic activity.

Multiplicity ratio calculations were performed as described in Hsia *et al*. [51]. Multiplicity refers to the ratio of action potential driven events (sEPSCs) to action potential independent events (mini EPSCs, or mEPSCs). If there is at most one synaptic contact with the recorded cell, then the multiplicity ratio will be 1. However, if there is more than one synaptic connection with the recorded cell the multiplicity will be increased, because the sEPSCs will have a larger amplitude than the mEPSCs, thus, the multiplicity ratio provides a measure of neuronal connectivity [52]. For multiplicity ratio measurements sEPSCs were recorded in the presence of aCSF with an elevated Ca^2+^/Mg^2+^ ratio to increase probability of release (aCSF composition (in mM): 125 NaCl, 2 KCl, 1.131 NaH_2_PO_4_, 26 NaHCO_3_, 4 CaCl_2_, 0.5 MgSO_4_ and 0.4 ascorbic acid), while in the presence of PTX. mEPSCs were recorded in the presence of PTX and tetrodotoxin (TTX) (0.5 μM)

### Evoked EPSCs

20 rats (5 males and 5 females from each prenatal treatment condition) were used to measure evoked glutamatergic EPSCs. Layer II/III pyramidal neurons were patched in the whole-cell configuration, superfused with Mg^2+^ free aCSF (CaCl_2_ concentration increased to 3mM), and cells allowed to equilibrate for 5 minutes in the presence of PTX (50 μM) before currents were evoked with a concentric bipolar stimulating electrode (FHC, Bowdoin ME) placed 100–200 μm away from the recording electrode in the apical dendritic field of the patched cell. Stimulation intensity was adjusted so that evoked currents had an average amplitude of ~500 pA. Initially, 8–10 ionontropic glutamate receptor-mediated EPSCs were recorded at 30 s intervals. To pharmacologically isolate NMDAR-mediated currents, PTX (50 μM) and NBQX (10 μM) were applied for 5 minutes before recording 8–10 evoked currents at 30 s intervals. GluN2B contribution to NMDA currents was then measured by blocking GluN2B containing NMDARs by applying ifenprodil (3 μM) for 5 minutes, after which 8–10 evoked currents were recorded at 30 s intervals. After ifenprodil treatment, residual NMDAR-mediated EPSCs were blocked by applying AP5 (50 μM) for 5 minutes before 8–10 evoked currents were recorded. Data were analyzed with Clampfit-10 (Molecular Devices, Sunnyvale, CA). Evoked current responses for each treatment were averaged and the peak evoked current amplitude measured. To measure AMPA contribution to total ionotropic glutamate receptor-mediated EPSCs, NMDA current traces were subtracted from traces of EPSCs recorded in the presence of PTX, and peak amplitudes calculated for both AMPA and NMDA mediated EPSCs. This information was then used to calculate AMPA/NMDA ratios.

### Biocytin Labeling

During electrophysiological recordings patched neurons were passively filled with 0.5% biocytin. After recordings were concluded brain slices were incubated in 4% paraformaldehyde for 24 hours at 4°C, then incubated in Tris-buffered saline (TBS) (500 mM Tris-HCl, 1.5 M NaCl) for 24 hours at 4°C. Brain slices were then placed in a TBS solution containing 0.5% Triton X-100 and 0.1% streptavidin-Cy3 for 1 hour in the dark at room temperature. The sections were then rinsed for 15 minutes with TBS before being mounted on slides and coverslipped with VECTASHIELD hard-set mounting media (Vector Laboratories, Burlingame, CA). Biocytin-labeled neurons were visualized on an Olympus BX51 microscope and imaged with an Olympus DP70 camera (Olympus, Center Valley, PA). Neurons that were confirmed to be layer II/III pyramidal neurons based on distinctive morphological characteristics (single apical field with a major branch point > 100 μm from the soma) were retained for analysis, and all other neuronal subtypes were excluded. It is also noted that selection of neurons from deeper layers did not occur as these are situated far from the area where neurons were selected for patching.

### Statistical Analyses

Statistical analyses were performed using SPSS ver. 21 for Macintosh (IBM, Armonk, NY). An alpha of p < 0.05 was adopted for all omnibus analyses. Effect sizes (partial eta squared,$\eta_{p}^{2}$) are provided for all significant effects. All data are presented as mean + 1 S.E.M. Autoradiography binding data for each radiolagand were analyzed separately using the multivariate analysis of variance (MANOVA) approach to repeated measures, with region (AID, LO, PrL, M1) as a within-subject factor and prenatal treatment (Saccharin, Ethanol) and sex as between-subject factors. For characterization of EPSC waveforms we conducted separate univariate analyses of variance (ANOVAs) for each measure of waveform characteristics (amplitude, rise time, area, half-width, tau1, tau2, frequency, multiplicity ratio) as dependent variables and prenatal treatment and sex as fixed factors. Ifenprodil sensitivity was analyzed with a univariate ANOVA, with prenatal treatment condition and sex as fixed factors.

## Results

### Voluntary drinking paradigm

Maternal serum ethanol levels, as well as other effects of the voluntary drinking paradigm’s effects on rat dams and their offspring are presented in **Table 1**. There was no significant effect of prenatal treatment condition on maternal weight gain during pregnancy, litter size, or pup birth weight.

**Table 1:** Effects of daily four-hour consumption of 5% ethanol on rat dams and their offspring.

	Saccharin Control	5% Ethanol
**Daily four-hour ethanol consumption**	NA	2.04(0.08) n = 32
(grams EtOH consumed/kg body weight/day)		
Daily four-hour ethanol consumption: Week 1	NA	1.73(0.09) n = 32
Daily four-hour ethanol consumption: Week 2	NA	2.07(0.09) n = 32
Daily four-hour ethanol consumption: Week 3	NA	2.04(0.08) n = 32
**Maternal serum EtOH concentration**	NA	60.8(5.8) n = 62
(mg EtOH/dL serum, 45 minutes into drinking)		
**Maternal weight gain during pregnancy**	107(4) n = 32	104(5) n = 32
(grams increase in body weight through GD20)		
**Litter size**	12.5(0.15) n = 32	12.2(0.29) n = 32
(number of live fetal pups/litter)		
**Pup birth weight**	6.17(0.13) n = 41	6.13(0.11) n = 42
(grams)		

Data are mean (SEM) with group sample size.

### Autoradiography analysis of ligand binding to glutamatergic receptors

Binding data in AID, LO, PrL, and M1 for each combination of prenatal treatment and sex are represented in **Table 2**. An example image of ifenprodil binding as well as non-specific binding is shown in **Fig. 1**. There were no significant main effects of prenatal treatment for any ligands [all ps > 0.44], however, there was a significant Region X Prenatal Treatment interaction for specific [^3^H]-ifenprodil binding [Wilks’ Λ = 0.638, F(3, 22) = 4.16, p = 0.018, $\eta_{p}^{2}$ = 0.362]. To characterize this interaction we first evaluated the pattern of prenatal treatment effects within each region. Inspection of the mean specific [^3^H]-ifenprodil binding in Table 2 indicates an approximately 12% increase in specific [^3^H]-ifenprodil binding within the AID region of PAE rats relative to saccharin controls which approached significance [F(1, 26) = 3.82, p = 0.057, $\eta_{p}^{2}$ = 0.133] compared to slight numerical reductions in specific [^3^H]-ifenprodil binding relative to saccharin controls in LO, PrL and M1 [all ps > 0.75]. This pattern suggests that the interaction was attributable to PAE-related increases in specific [^3^H]-ifenprodil binding in AID, compared to modest PAE-related decreases in other regions. Evaluation of regional variation in PAE effects is, however, complicated by large regional variation in specific [^3^H]-ifenprodil binding independent of prenatal treatment. Therefore, to more clearly characterize the nature of this interaction, specific [^3^H]-ifenprodil binding values for PAE rats relative to control values were calculated (PAE value / saccharin control value) within each region (see **Fig. 1D**). Because the sex factor was not involved in this interaction the data were collapsed across levels of sex, however, we note that the outcome of the contrast was comparable for both sexes and exclusion of the sex factor did not alter the result of this analysis. The resulting values were analyzed using repeated measures ANOVA with region as a single factor, and a contrast to compare the ethanol-related change in specific [^3^H]-ifenprodil binding for AID to the other three regions. This contrast was significant [F(1, 13) = 13.79, p < 0.003, $\eta_{p}^{2}$ = 0.515] and accounted for >99% of regional variation in the effects of PAE on specific [^3^H]-ifenprodil binding among a set of orthogonal contrasts, further suggesting that the Prenatal Treatment X Region interaction was attributable to a pattern of increased specific [^3^H]-ifenprodil binding in AID compared to decreases observed in the other regions. No significant Region X Prenatal Treatment interactions were observed for the specific binding of the other radioligands [all ps > 0.45] and no other two- or three-way interactions were significant for any radioligands [all ps > 0.056]. There was a significant effect of sex for MK801 binding [female > male; F(1, 24) = 27.40, p < 0.001, $\eta_{p}^{2}$ = 0.133]. No other sex main effects were significant [all ps > 0.09]. There were also significant main effects of region for each ligand (all ps < 0.001; see **Table 2**) that are not further characterized here.

**Table 2:** Effect of prenatal ethanol exposure on the density of various glutamate receptor subtypes in frontal cortex.

	AID		LO		PrL		M1	
Tritiated Radioligand	SAC	ETOH	SAC	ETOH	SAC	ETOH	SAC	ETOH
**AMPA Receptor**	**3.11(0.08)**	**3.09(0.07)**	**2.30(0.07)**	**2.22(0.06)**	**3.50(0.07)**	**3.41(0.07)**	**2.02(0.05)**	**2.00(0.07)**
**Fluorowillardiine** *[R]								
*Male*	*3.12(0.13)*	*3.09(0.11)*	*2.26(0.13)*	*2.16(0.07)*	*3.53(0.11)*	*3.47(0.09)*	*1.91(0.05)*	*2.07(0.09)*
*Female*	*3.10(0.12)*	*3.08(0.10)*	*2.34(0.07)*	*2.28(0.10)*	*3.48(0.10)*	*3.36(0.12)*	*2.13(0.07)*	*1.94(0.11)*
**NMDA Receptor**	**0.61(0.04)**	**0.60(0.03)**	**0.47(0.03)**	**0.48(0.02)**	**0.50(0.03)**	**0.45(0.03)**	**0.55(0.03)**	**0.54(0.03)**
**MK-801** *[R, S]								
*Male*	*0.52(0.04)*	*0.54(0.03)*	*0.41(0.02)*	*0.43(0.02)*	*0.42(0.03)*	*0.38(0.03)*	*0.51(0.02)*	*0.46(0.03)*
*Female*	*0.69(0.05)*	*0.67(0.04)*	*0.54(0.03)*	*0.52(0.03)*	*0.57(0.03)*	*0.52(0.02)*	*0.58(0.06)*	*0.61(0.03)*
**GluN2B**	**0.45(0.02)**	**0.51(0.02)**	**0.42(0.02)**	**0.41(0.02)**	**0.49(0.03)**	**0.48(0.03)**	**0.34(0.02)**	**0.33(0.02)**
**Ifenprodil** *[R, PTxR]								
*Male*	*0.44(0.04)*	*0.49(0.02)*	*0.43(0.04)*	*0.40(0.03)*	*0.51(0.05)*	*0.51(0.05)*	*0.32(0.03)*	*0.33(0.02)*
*Female*	*0.46(0.02)*	*0.52(0.03)*	*0.41(0.01)*	*0.43(0.03)*	*0.47(0.05)*	*0.44(0.04)*	*0.36(0.01)*	*0.34(0.03)*
**mGluR**_**2,3**_	**6.35(0.25)**	**6.09(0.25)**	**6.12(0.26)**	**5.88(0.24)**	**5.84(0.25)**	**5.52(0.26)**	**6.93(0.26)**	**6.66(0.27)**
**LY-341495** *[R]								
*Male*	*6.54(0.38)*	*6.47(0.39)*	*6.27(0.42)*	*6.22(0.33)*	*6.07(0.37)*	*5.93(0.39)*	*7.21(0.38)*	*7.11(0.37)*
*Female*	*6.15(0.34)*	*5.71(0.28)*	*5.98(0.33)*	*5.53(0.32)*	*5.61(0.35)*	*5.11(0.29)*	*6.66(0.35)*	*6.21(0.33)*
**MGluR**_**5**_	**0.55(0.02)**	**0.55(0.01)**	**0.45(0.01)**	**0.44(0.01)**	**0.69(0.01)**	**0.68(0.02)**	**0.45(0.01)**	**0.43(0.01)**
**MPEPy** *[R]								
*Male*	*0.55(0.02)*	*0.53(0.01)*	*0.45(0.02)*	*0.43(0.01)*	*0.68(0.01)*	*0.66(0.03)*	*0.43(0.01)*	*0.41(0.01)*
*Female*	*0.55(0.03)*	*0.57(0.02)*	*0.44(0.01)*	*0.46(0.01)*	*0.70(0.02)*	*0.69(0.02)*	*0.47(0.01)*	*0.44(0.01)*

Data are the mean (SEM) specific binding, expressed as femtomoles bound / 10^5^ μm^2^, for each radioligand for the total sample (in bold, N = 14) and separately for males (n = 7 per prenatal treatment) and females (n = 7 per prenatal treatment). R = Region, PT = Prenatal Treatment, S = Sex.

* indicates a significant effect at p < 0.05; See Fig. 1D for additional data relevant to characterization of the PTxR interaction for specific [^3^H]-ifenprodil binding.

### Characterization of sEPSCs and mEPSCs from AID layer II/III pyramidal neurons

**Fig 2:**
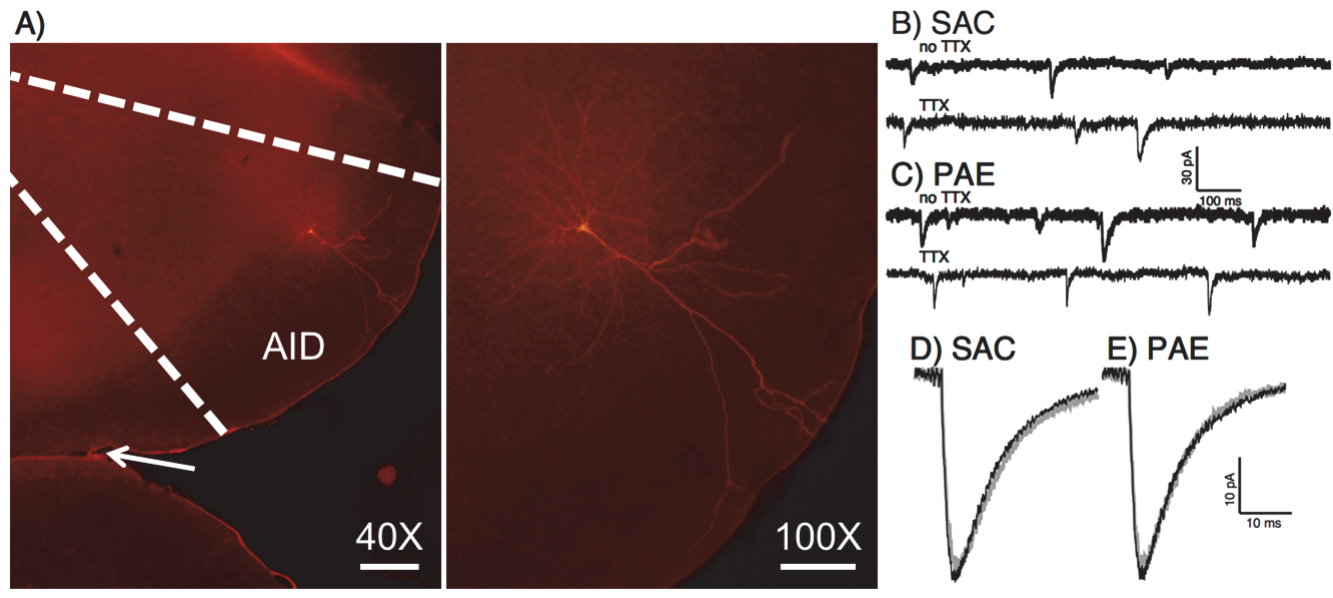
Sample layer II/III pyramidal neuron and spontaneous current recordings. A) Example of biocytin-filled pyramidal neuron from cortical layer II/III. The internal solution for whole-cell patch-clamp electrophysiology contained biocytin to visualize patched neurons in paraformaldehyde fixed slices using fluorescence microscopy with streptavidin conjugated Cy3. A low magnification (40X) image is shown delineating agranular insular cortex, along with a higher magnification image (100X) to show neuronal morphology. The arrow in the 40X image points to the rhinal fissure. Scale bar 40X = 200 μm, scale bar 100X = 100 μm. B-E) Sample traces and average waveforms of sEPSCs and mEPSCs from AID layer II/III pyramidal neurons. Sample traces from saccharin exposed (B) and PAE (C) animals with sEPSC traces (no TTX) and mEPSC (TTX) traces are shown. Average waveforms for both sEPSCs and mEPSCs from saccharin exposed (D) or PAE (E) animals are also displayed. There were no significant effects of prenatal treatment condition on EPSCs.

sEPSC and mEPSC waveform characteristics were collected from layer II/III pyramidal neurons within AID. The internal solution used for whole-cell patch-clamp electrophysiology contained biocytin to confirm that measurements were taken from pyramidal neurons. An example of a biocytin-stained pyramidal neuron from AID is presented in **Fig. 2A**. Average waveform characteristics for both sEPSCs and mEPSCs fit with a dual exponent function, as well as multiplicity ratio data are presented in **Table 3**. Sample traces and average waveforms for both sEPSCs and mEPSCs are shown in **Fig. 2B-E**. There were no significant effects of prenatal treatment on any of the waveform characteristics or multiplicity ratios. However, there were significant main effects of sex for several waveform characteristics: amplitude for both sEPSCs [male > female; F(1, 18) = 64.66, p < 0.001, $\eta_{p}^{2}$ = 0.802] and mEPSCs [male > female; F(1,16) = 44.702, p <0.001, $\eta_{p}^{2}$ = 0.736], half-width for sEPSCs [female > male; F(1,16) = 4.935, p = 0.041, $\eta_{p}^{2}$ = 0.236, tau1 for both sEPSCs [female > male; F(1,16) = 4.626, p = 0.047, $\eta_{p}^{2}$ = 0.224] and mEPSCs [female > male; F(1,16) = 8.949, p = 0.009, $\eta_{p}^{2}$ = 0.359], and tau2 for mEPSCs [female > male; F(1,16) = 9.044, p = 0.008, $\eta_{p}^{2}$ = 0.361].

**Table 3:** Characteristics of average sEPSC and mEPSC waveforms fit with a dual exponent function (A) and multiplicity ratio data (B).

	sEPSC		mEPSC	
A)	SAC	PAE	SAC	PAE
**Amplitude (pA)** ***[s,m]	**−38.53(1.31)**	**−38.15(1.55)**	**−37.64(1.38)**	**−36.93(1.65)**
*Male*	*−42.27(0.44)*	*−42.11(1.60)*	*−40.79(0.94)*	*−41.50(1.10)*
*Female*	*−34.79(0.75)*	*−34.18(0.61)*	*−34.48(1.64)*	*−32.35(0.75)*
**Rise time (ms)**	**1.80(0.13)**	**1.76(0.11)**	**2.38(0.32)**	**2.01(0.17)**
*Male*	*1.73(0.11)*	*1.64(0.16)*	*1.92(0.26)*	*1.79(0.10)*
*Female*	*1.87(0.25)*	*1.87(0.15)*	*2.84(0.53)*	*2.24(0.31)*
**Area (pa/ms)**	**−705.46(61.62)**	**−620.63(78.15)**	**−707.85(64.57)**	**−608.58(58.08)**
*Male*	*−695.90(64.67)*	*−683.63(96.01)*	*−728.79(67.45)*	*684.44(104.20)*
*Female*	*715.03(113.39)*	*557.62(127.59)*	*686.92(118.29)*	*−532.72(37.99)*
**Half-width (ms)** *[s]	**8.08(0.66)**	**7.52(0.80)**	**8.52(0.78)**	**7.43(0.56)**
*Male*	*7.27(0.56)*	*6.20(0.57)*	*7.15(0.80)*	*7.05(0.50)*
*Female*	*8.88(1.14)*	*8.84(1.31)*	*9.88(1.08)*	*7.80(1.05)*
**Tau1 (ms)** * [s], ** [m]	**6.42(0.76)**	**6.89(0.97)**	**7.57(0.91)**	**6.43(0.68)**
*Male*	*5.43(0.60)*	*5.42(0.85)*	*5.58(0.69)*	*5.64(0.75)*
*Female*	*7.40(1.31)*	*8.37(1.57)*	*9.57(1.12)*	*7.22(1.09)*
**Tau2 (ms)** **[m]	**15.06(2.08)**	**14.91(3.17)**	**15.95(1.55)**	**13.30(1.18)**
*Male*	*13.59(1.99)*	*9.30(1.26)*	*13.15(1.65)*	*11.14(1.12)*
*Female*	*16.54(3.80)*	*20.53(5.28)*	*18.76(2.05)*	*15.46(1.64)*
**Frequency (Hz)**	**0.22(0.04)**	**0.18(0.04)**	**0.13(0.03)**	**0.16(0.04)**
*Male*	*0.18(0.04)*	*0.15(0.03)*	*0.15(0.05)*	*0.22(0.05)*
*Female*	*0.25(0.07)*	*0.22(0.08)*	*0.10(0.02)*	*0.10(0.05)*
**B)**	SAC	PAE		
**Multiplicity**	**1.04(0.07)**	**1.06(0.04)**		
*Male*	*0.99(0.12)*	*1.02(0.06)*		
*Female*	*1.09(0.06)*	*1.11(0.05)*		

Data are mean (SEM) for each characteristic for the total sample (N = 10 animals per prenatal treatment) and separately for males (n = 5 animals per prenatal treatment) and females (n = 5 per prenatal treatment). There were no significant main effects of prenatal treatment or prenatal treatment X sex interactions at p < 0.05.

*indicates a significant effect at p<0.05 for **sex main effects** for either the sEPSC[s] or mEPSC[m].

** p<0.01

***p<0.001

### Analysis of evoked glutamatergic EPSCs

**Fig 3:**
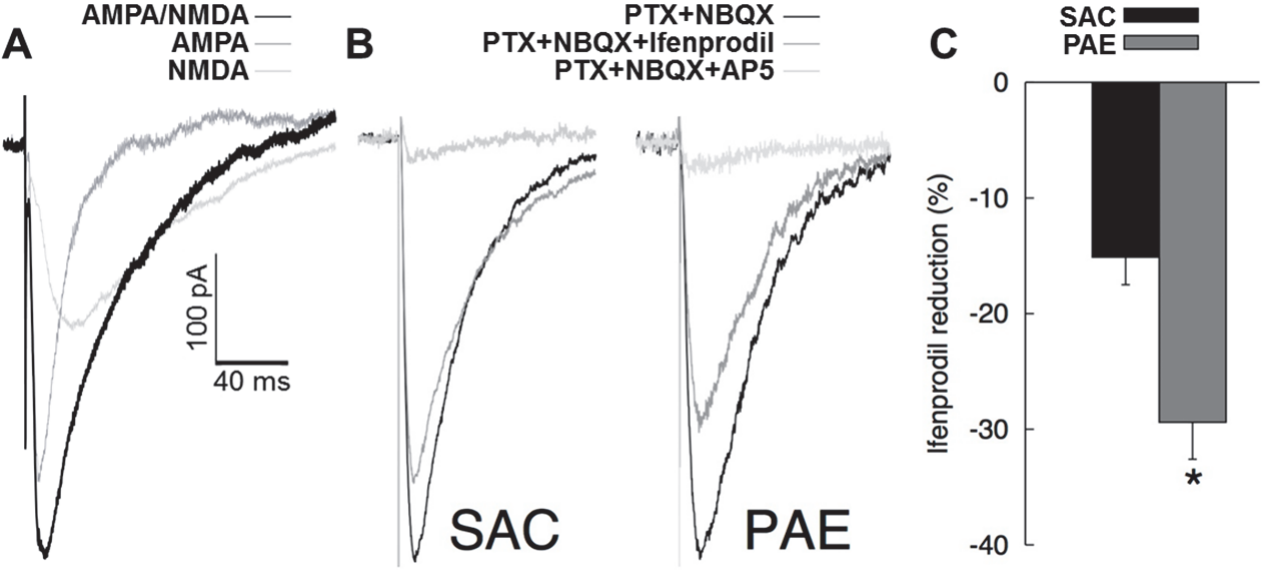
Evoked currents and ifenprodil sensitivity. A) Representative glutamatergic EPSCs. Representative traces from a combined AMPA/NMDA EPSC, an NMDA-mediated EPSC, and an AMPA-mediated EPSC calculated by subtracting the NMDA EPSC from the combined EPSC are shown. B) Representative evoked NMDA EPSCs. Sample traces of evoked NMDA EPSCs from both prenatal treatment conditions with amplitudes of total NMDA currents normalized between treatment conditions. C) Sensitivity of evoked NMDA EPSCs to ifenprodil. Mean percent (+SEM) reduction in evoked NMDA current following application of 3μM ifenprodil (n = 10 per prenatal treatment). Asterisk (*) indicates a significant effect (p = 0.002) of prenatal treatment condition on ifenprodil sensitivity.

A representative ionotropic glutamatergic EPSC, as well as an AMPA-mediated and NMDA-mediated EPSC are shown in **Fig. 3A**. No effect of prenatal treatment condition on evoked AMPA/NMDA current amplitude ratios was detected [F(1,16) = 0.07, p = .795] (**Table 4**). There was no effect of sex or Sex X Prenatal treatment on AMPA/NMDA ratios (both ps > 0.56).

**Table 4:** AMPA/NMDA ratios.

	SAC	PAE
**AMPA/NMDA**	**1.84(0.26)**	**1.75(0.21)**
*Male*	*1.91(0.22)*	*1.88(0.41)*
*Female*	*1.77(0.51)*	*1.61(0.14)*

Data presented are mean (SEM) AMPA/NMDA ratios for the total sample (N = 10 animals per prenatal treatment) and separately for males (n = 5 per prenatal treatment) and females (n = 5 per prenatal treatment).

Representative evoked responses obtained at baseline (PTX+NBQX), in the presence of ifenprodil and in the presence of AP5 are shown in **Fig. 3B**. Mean percent reductions in evoked responses with 3μM ifenprodil for each prenatal treatment condition are shown in **Fig. 3C**. There was a significant effect of prenatal treatment on percent reduction in evoked response amplitude [PAE > SAC; F(1, 16) = 14.28, p = 0.002 $\eta_{p}^{2}$ = 0.47]. The main effect of sex and the Sex X Prenatal Treatment interaction were not significant [both *p*s > 0.38]. We note that the effects of prenatal treatment on ifenprodil sensitivity were comparable for males and females (both ps < 0.032). Mean (SEM) % reduction to evoked NMDA EPSCs following ifenprodil application broken down by sex are as follows: SAC_male_ = -16.50(3.16), PAE_male_ = -32.60(5.33); SAC_female_ = -15.00(3.42), PAE_female_ = -27.34(2.55).

## Discussion

In order to better understand the impacts of PAE on excitatory neurotransmission in the frontal cortex, initial experiments in this study focused on autoradiography analysis of radioligand binding to glutamatergic neurotransmitter receptors in layer II/III pyramidal neurons in the frontal cortex. PAE did not have a significant effect on ligand binding to total NMDA, AMPA, mGluR_2/3,_ or mGluR_5_ receptors in the regions examined (AID, LO, PrL, M1). [^3^H]-Ifenprodil binding to GluN2B-containing NMDARs was affected by PAE with increased [^3^H]-ifenprodil binding in cortical layers II/III of AID compared to modest decreases in binding in PrL, LO and M1. Motivated by this observation subsequent examination of AMPA and NMDA receptor physiology in layer II/III pyramidal neurons of AID yielded evidence of enhanced sensitivity to the GluN2B antagonist ifenprodil following PAE, whereas no effects of PAE on AMPA receptor sEPSCs and mEPSCs, multiplicity ratios (as a measure of neuronal connectivity [51]), or AMPAR/NMDAR ratios were observed. Collectively these observations suggest that AMPAR expression and function in frontal cortex are spared following PAE, whereas expression and function of GluN2B-containing subunits are enhanced selectively in the AID region of frontal cortex.

Altered NMDAR composition in AID caused by PAE could help to explain observations of PAE-related alterations in AID function and AID-dependent behavioral and cognitive processes previously reported by our laboratory [34, 35, 53]. Among these are changes in social behaviors frequently observed following PAE, which prior work from our laboratory has linked to AID and adjacent ventrolateral frontal cortex. Hamilton *et al*. [53] recently demonstrated that PAE significantly increased the frequency and duration of wrestling in adult rats, suggesting increased aggression, while simultaneously decreasing social investigation (e.g., anogenital sniffing). These results replicated data from a previous study within our laboratory [35]. Modifications in social behavior following PAE have also been reported by other laboratories using a variety of experimental conditions including: dosage, timing and duration of exposure, age at time of measurement, and exposure methods [54–59]. In addition to PAE-induced alterations in social behavior, Hamilton *et al*. [53] showed PAE also impairs tongue protrusion, which critically depends upon ventrolateral frontal cortex regions including AID [60, 61] as well as response perseveration errors during spatial reversal learning [62]. Given these observations it is interesting to speculate that altered NMDAR subunit composition in AID may contribute to PAE-induced alterations in AID-dependent behaviors. Increased association of PSD-95 with GluN2B in both cortex and hippocampus correlates with age-related declines in reversal learning [63]. Genetic manipulations that increase GluN2B and reduce GluN2A expression in the forebrain enhance and impair social recognition memory, respectively [64, 65], while other manipulations of NMDAR function impact other measures of social behavior [66–68]. Based on these observations, it is possible that the altered NMDAR subunit expression and function seen in this study underlie some aspects of social behavior alterations caused by PAE previously observed by our laboratory, however, the relationship between the present observations and behavioral consequences of PAE require examination in future studies. Considering that orbitofrontal cortex, of which AID is a component, has been linked to reward processing in a number of different contexts and species[69–71], it is also tempting to speculate that enhanced GluN2BRs in AID could contribute to increased voluntary ethanol consumption following PAE, which has been reported following a variety of PAE paradigms [46, 72–74], including the one utilized in the current study [75]. Evaluation of this possibility awaits further study.

The present findings join a growing body of data demonstrating that ethanol exposure during development influences NMDAR expression and subunit composition. Several studies have reported reductions in NMDAR binding in cortex and hippocampus [76–78] following PAE. Characterization of specific NMDAR subunit expression following PAE has, however, yielded more varied results. Several independent laboratories have reported reduced GluN2B expression in whole brain [79, 80], cortex [81–83], and hippocampus [82], as well as reduced PSD-95 associated GluN2B [84] or synaptosomal GluN2B [85] in hippocampus following PAE. In contrast, some studies have failed to detect effects of PAE on GluN2B expression in cortex [86–88] or hippocampus [89, 90]. Consistent with the findings of the present study, one study reported a significant increase in cortical GluN2B mRNA expression [91], and another yielded non-significant trends for increases in cortical GluN2B protein in response to PAE [90]. These conflicting characterizations of GluN2B expression following PAE may be partially attributed to differences in the dose, duration, timing, age at time of exposure, age at time of measurement, species examined, brain region examined, and other variables. It is worth noting that to our knowledge, this study represents the first report of glutamatergic receptor expression in individual fronto-cortical subregions following PAE.

The effects of ethanol exposure during development are not limited to ventrolateral frontal cortex, as other fronto-cortical subregions are affected by a prenatal ethanol insult. Studies examining medial prefrontal cortex (mPFC) have found PAE induced alterations in gene expression [34, 35, 72, 92, 93], dendritic morphology [94] and mPFC-dependent behaviors [93, 95]. AID has reciprocal connections to mPFC [96], so circuit-level effects of PAE are likely. Further, previous work from our laboratory has observed PAE-dependent changes in dendritic morphology in the nucleus accumbens [97], a region that receives efferent projections from AID [98]. Understanding how PAE impacts neuronal activity in interconnected brain regions will provide insight into how this teratogen affects brain development at a circuit-level, and could lead to beneficial treatments by enhancing or inhibiting neuronal activity in a particular region to rescue deficits in activity in associated areas.

Several limitations of the present study and findings warrant discussion. That total NMDAR measures were not affected by PAE, but measures of GluN2BR expression and function were enhanced by PAE, suggests decreases in other subunits such as GluN2ARs. We examined binding and evoked currents for total NMDARs and GluN2BRs, but did not examine other subunits based on currently available pharmacological agents to target these receptors. For example, NVP-AAM077 has been utilized in a number of studies as a GluN2A specific antagonist, but recent examinations of binding specificity have demonstrated that it has mixed affinity for GluN1/GluN2A and GluN1/GluN2B NMDA receptors [99], therefore it could not be utilized to generate unambiguous radioligand binding or electrophysiological data. It is also important to note that the electrophysiological data on GluN2B function were more robust than the autoradiography data, which in part may reflect the specificity of the measures. Following observations of increased GluN2B binding in AID we also analyzed brain punches from AID and observed no overall differences in GluN2A and GluN2B protein expression following PAE, which was likely due to the diversity of cell types included in the brain punch. Analyses targeting superficial layers revealed increased binding (autoradiography) and selective analysis of layer II/III pyramidal neurons in AID yielded evidence of increased ifenprodil sensitivity. Collectively, these observations suggest that PAE-related enhancements of GluN2B expression and function in AID are likely most robust in superficial pyramidal neurons. Another limitation in the present study concerns potential stressors in dams during the alcohol exposure paradigm. Dams are housed in isolation during the pre-pregnancy and pregnancy drinking phases. This could increase stress, however, it is noted that dams from both prenatal exposure conditions are isolated in the same manner. This procedure was utilized to allow accurate measurement of maternal alcohol consumption, to match procedures utilized in our prior studies. Finally, the use of the non-competitive NMDA antagonist ketamine as an anesthetic prior to decapitation for acute slice recordings of NMDA currents warrants discussion. Ketamine was utilized to reduce the potential for excitotoxic damage during and following decapitation. Healthy, viable slices can be obtained using this approach [100], although there is potential for residual ketamine to remain bound to the NMDA receptors after slice preparation. This could result in underestimation of evoked NMDA current amplitudes, however, this is unlikely as slices were extensively washed in ketamine-free aCSF during both recovery and perfusion in the recording chamber. Accordingly, we have not encountered problems with ketamine in previous studies examining NMDA receptor currents in brain slices (see [101]), and evoked NMDA responses in our studies are comparable to those reported in the literature examining neighboring cortical areas [102].

In this study, [^3^H]-Ifenprodil binding in AID and sensitivity of layer II/III AID pyramidal neurons to ifenprodil was similar in both sexes, indicating that GluN2B expression in response to PAE is independent of sex. We also note that all four stages of estrous are represented equally in both prenatal treatment conditions for these data. While the sample sizes are too small to support a formal analysis of the contribution of stage of estrous to the observed results, the uniform representation of estrous stages in each treatment condition and the lack of a sex effect suggest that effects of PAE on GluN2BR currents are not critically related to variation with estrous. There are, however, a number of studies demonstrating sexually dimorphic effects of prenatal ethanol exposure [35, 54, 103–110], so it is possible that other aspects of glutamatergic receptor expression or channel physiology may be affected by PAE differentially in the two sexes. A particularly interesting observation in this study concerns fundamental differences in AMPAR electrophysiology between males and females. AMPAR-mediated EPSCs from male animals displayed larger amplitudes, shorter half-widths, and faster decay times than EPSCs from female animals (**Table 2**), characteristics that were not altered by prenatal treatment condition. Sex differences in AMPAR expression and physiology are not without precedent, as sex differences in AMPAR subunit expression [111] and EPSP amplitude [112] have been observed in the hippocampus. Sex-dependent divergences in AMPAR channel physiology may underlie differences in AID-dependent social behaviors between males and females previously observed [35], and are worth investigating further.

Several avenues for future research are motivated by the findings of the present study. Increased GluN2B expression in AID could have a number of functional consequences, as NMDAR subunit composition determines the NMDA receptor’s electrophysiological properties. Antagonism, knock-out, or siRNA knock-down of GluN2BRs impairs LTP in anterior cingulate cortex [113] and hippocampus [114–116], although some data suggest an important role of GluN2BRs in long-term depression (LTD) [117–119]. At present little information exists about the role of GluN2B in synaptic plasticity in frontal cortex, however, the available data from other circuits suggest that the increased GluN2B expression observed in this study following PAE should facilitate LTP. This prediction stands in contrast to the LTP deficits observed in perforant path-dentate gyrus synapses following moderate PAE [120, 121]. This potential double dissociation, between the effects of PAE exposure on AID and hippocampal synaptic circuits with regard to GluN2B subunit expression, requires evaluation in future studies. NMDARs are heterotetrameric assemblies typically comprised of two obligatory channel-forming GluN1 subunits and any combination of regulatory GluN2 and GluN3 subunits (for review see [122]). In the forebrain GluN2A and GluN2B subunit expression dominates, with GluN2B expression peaking during early postnatal development [123]. During later postnatal development GluN2B expression decreases with a concurrent increase in GluN2A expression [124]. Thus, it will be important to extend the observations reported here in adult animals to receptor expression changes during development. Future studies are also needed to determine if the PAE-related enhancements of GluN2BRs in AID reported here represent a possible mechanism of PAE-related deficits in AID-dependent social behavior, motor behavior, and reversal learning[53]. In addition to examining relationships between behavior and GluN2BR currents in the slice preparation, analysis of AID-dependent behaviors and GluN2BR currents in non-exposed animals with viral overexpression of GluN2B in AID, similar to the approach utilized in ref. [125] for medial frontal cortex-dependent behaviors, would help further establish the links between behavior, physiology, and GluN2BRs in AID.

In summary, the combined data from this study demonstrate a region-specific enhancement of GluN2B containing NMDA receptor binding and GluN2B-dependent NMDAR activity in AID following developmental ethanol exposure using a voluntary exposure paradigm. These GluN2B expression changes following PAE may have consequences on AID-dependent behaviors.
